# The importance of school lunches to the overall dietary intake of children in Sweden: a nationally representative study

**DOI:** 10.1017/S1368980020000099

**Published:** 2020-07

**Authors:** Patricia Eustachio Colombo, Emma Patterson, Liselotte S Elinder, Anna Karin Lindroos

**Affiliations:** 1 Department of Global Public Health, Karolinska Institutet, 171 77 Stockholm, Sweden; 2 Centre for Epidemiology and Social Medicine, Region Stockholm, 104 31 Stockholm, Sweden; 3 The Swedish Food Agency, 751 26 Uppsala, Sweden; 4 Department of Internal Medicine and Clinical Nutrition, Institute of Medicine, Gothenburg University, 413 45 Gothenburg, Sweden

**Keywords:** Children, Adolescents, School meals, Health-promoting, Nutrient intake, Dietary habits, Socioeconomic status

## Abstract

**Objective:**

School lunches have potential to foster healthy diets in all children, but data on their importance are relatively scarce. The current study aimed to describe the dietary intake from school lunches by sex and school grade, and to assess how the daily intake, school lunch intake and the daily intake provided by lunch differ by sex and parental education.

**Design:**

Cross-sectional. All foods and drinks consumed for 1–3 weekdays were self-reported. Energy, absolute and energy-adjusted intakes of nutrients and food groups were calculated per weekday and per school lunch. Mixed-effects linear models assessed sociodemographic differences in dietary intakes. Nutrient and energy density at lunch and during the rest of the day were compared.

**Setting:**

Seventy-nine Swedish primary schools.

**Participants:**

Pupils in grades 5 and 8 (*N* 2002), nationally representative.

**Results:**

Lunch provided around half of daily vegetable intake and two-thirds of daily fish intake. Nutrient density was higher and energy density lower at lunch compared with the rest of the day (*P* < 0·001). Boys had greater energy-adjusted intakes of red/processed meat and lower intakes of vegetables and dietary fibre compared with girls (*P* < 0·001), overall and at lunch. Daily energy-adjusted intakes of most nutrients/food groups were lower for pupils of lower-educated parents compared with pupils of parents with higher education, but at lunch, only Fe and fibre intakes were significantly lower in this group.

**Conclusions:**

School lunches are making a positive contribution to the diets of Swedish children and may mitigate well-established sex differences and social inequalities in dietary intake.

Poor dietary habits account for a significant proportion of the global burden of disease, contributing to overweight and obesity and to the risk of non-communicable diseases^(1,2)^. It has been well established that there is a strong gradient in the prevalence of chronic diseases to the disadvantage of groups with low socioeconomic status^(3,4)^, and that part of this social inequality is associated with a lower diet quality^(5,6)^. For example, findings from the Nordic countries reveal social inequalities in food habits, with socioeconomically disadvantaged children being less likely to consume fruit and vegetables^(7)^. Sex differences in dietary intake have also been observed in Swedish adults^(8)^ and children^(9)^ as well as in other contexts with males consuming less fruit and vegetables compared with females^(10–12)^.

Enabling all children to establish healthy dietary habits early in life, and providing a supportive environment for a healthy diet, is therefore key to promoting public health^(13)^. School meals, particularly when free-of-charge or heavily subsidised, can reach children of every socioeconomic background and can make up a considerable proportion of children’s dietary intake over a long and critical period of growth^(14)^. They have thus a great potential to contribute to fostering healthy dietary habits in all children, thereby reducing social health inequalities in the long term^(15,16)^.

Children’s diets are determined by many socioeconomic and sociocultural factors, including the home^(17)^ and school environment^(18)^. School meals have been shown to play an important role in the food security of socioeconomically disadvantaged children in the USA^(19)^. In the UK^(20)^ and Denmark^(21)^, children’s food choices at lunchtime on school days have been shown to contribute significantly to the overall intake of healthy foods such as vegetables. School lunch consumption in Finland has further been shown to contribute positively to a healthy diet^(22)^ and a healthier overall eating pattern outside school^(23)^.

The characteristics of school meal services vary widely across the world; in high-income countries, this service is generally available for free or at a reduced price^(15)^. Countries like Estonia and Brazil have widespread school meal programmes, yet only Finland and Sweden provide lunches free-of-charge to all children in primary and lower secondary school, up to the age of 16, regardless of parental income^(15)^. In a previous study, intakes from Swedish school meals from 2003^(24)^ were compared with reference values used for planning school meals^(25)^ – 30 % of daily intakes recommended by the Nordic Nutrition Recommendations^(26)^. Children in grades 2 and 5 had lower mean intakes of dietary fibre, PUFA and vitamin D, but higher mean intakes of saturated fat. In the older age group, the mean Fe intake and mean folate intake was also lower compared with reference values.

Publicly financed school meals were introduced in both Finnish and Swedish contexts in the late 1940s^(27,28)^. The Swedish lunch typically consists of hot main dishes, a salad buffet, bread, spread and milk or water. In 2011, a new law was enacted in Sweden stating that school meals should be nutritious^(29)^, and since then, school meal quality has improved considerably^(30)^. However, the dietary intake from school lunches has not been assessed since 2003, and school lunch intake in children older than 11–12 years, across sex and socioeconomic groups, has never been assessed in Sweden. In 2016–2017, the Swedish Food Agency carried out a national dietary survey of children and adolescents in grades 5, 8 and 11 using a new and validated dietary assessment method^(31)^. This new data presents a unique opportunity to re-assess the contribution that school meals make to children’s total diet and to explore their potential in promoting healthy dietary habits and social equality. The aim of the current study was to (a) describe the intake of foods and nutrients from school lunches by sex and school grade, and (b) assess how the overall daily intake, school lunch intake and the contribution from school lunches to the overall daily intake differ by sex and parental education.

## Methods

### Design

The current study was based on data from the cross-sectional Swedish dietary survey Riksmaten Adolescents 2016–2017. A total of 601 schools were selected by Statistics Sweden to provide a nationally representative sample of Swedish pupils in school grades 5, 8 and 11. The schools were sampled based on municipality types, type of school (publicly/independently run school) and geographical spread. Trained assistants performed school visits to collect dietary data through a validated web-based dietary assessment method from August 2016 to June 2017. A more detailed description of the survey and methods has been presented elsewhere^(32)^.

### Participants

Overall, 131 schools agreed to participate, 5145 pupils were invited to take part, and 3477 pupils participated in at least one part of the survey^(32)^, with 2968 providing complete dietary information for 3 d. The distribution on school level was skewed towards larger schools, but was representative in terms of type of municipality and reflected the distribution of public and independent schools in Sweden^(32)^. Pupils in grade 11 (*n* 966) were excluded since the free meal entitlement only applies to the compulsory (9-year) school system. This resulted in a final study sample of 2002 pupils (grade 5 = 990 pupils; grade 8 = 1012 pupils) from seventy-nine schools.

### Data and variables

Dietary intake and other relevant information was self-reported through RiksmatenFlex, which includes a validated dietary registration part and web questionnaires^(31)^. The types and amounts of all foods and drinks consumed were recorded by the pupils for two consecutive days and a third separate day in the dietary registration tool. The first day was the day before the school visit and was recorded retrospectively as a 24-h recall on the day of the visit. The second day was recorded during the day of school visit, and the third day, also recorded using a 24-h recall, was randomly assigned 4–10 d after day 1. Energy and nutrient intakes were calculated automatically by linkage with the Swedish Food Agency’s food composition database, Riksmaten Adolescents 2016–2017. Dietary supplements were not included in the nutrient calculations.

#### Dietary intake

For the purpose of the current study, only intakes on school days were included in the analyses. The study population (2002 pupils in grades 5 and 8) had complete dietary intake data for 3 d including non-school days (i.e. 6006 d). After exclusion of non-school days (*n* 1241) and days when a lunch was eaten but it was neither a ‘school lunch’ nor eaten on the school premises (*n* 667), 4098 d were available, with each pupil contributing to 2 (*n* 1241), 3 (*n* 760) or 1 (*n* 1) d of dietary intake data. The mean (overall) daily intake, mean intake at (school) lunch and mean intake excluding lunch (i.e. intake during the rest of the day) was calculated for each individual.

The current study focuses on intakes of energy and vitamin D, Fe, folate, dietary fibre and saturated fat. The first five have previously been found to be present in school meals in amounts lower than the reference values to be used for planning school meals, while the intake of saturated fat has been found to be higher^(24)^. Food groups such as vegetables, fish (both oily and white fish) and red/processed meat were also analysed as they are included in the Swedish food-based dietary guidelines^(33)^. Intakes of a food group included both amounts recorded directly and amounts estimated from dishes. The absolute intake (µg, mg or g of nutrients or food group) as well as the energy-adjusted intake (µg, mg or g of nutrients or food group per 10 MJ) at lunch and during the rest of the day, respectively, was calculated. Intake at lunch was also expressed as a percentage of total daily intake. Energy density at lunch and during the rest of the day was also calculated since this has been suggested to be a marker of diet quality in Swedish adolescents^(34)^. This was done by dividing the total energy intake (kJ) from food (including soups and yoghurt) by the total weight of food, excluding drinks, as suggested previously^(34,35)^. All intakes are energy-adjusted unless otherwise specified.

#### Sociodemographic variables and anthropometrics

Information on sex, age and school grade was available before the school visit. Parents’ highest level of education attained (categorised as >12 or ≤12 years of schooling) was reported by a parent in a separate questionnaire. If the pupil and at least one parent was born in one of the Nordic countries (Sweden, Finland, Norway, Denmark or Iceland), that subject was then classified as Nordic. The same classification was applied to pupils who were born outside of the Nordic countries but where both parents were Nordic-born. All others were considered non-Nordic. Height and weight were measured during the school visit using standardised methods^(36)^ by trained assistants and calibrated equipment. Overweight and obesity was defined according to the International Obesity Task Force BMI cut-offs for children^(37)^. The area of residence was categorised as urban (large cities/municipalities close to large cities), semi-urban (larger cities/municipalities close to larger cities) or rural (smaller cities/densely populated areas) based on the data from Statistics Sweden^(32)^.

### Statistical analyses

For all statistical computations, the software R version 3.4.3 was used^(38)^. All intakes were calculated and presented as means with 95 % CI or sd using embedded functions as well as the R packages ‘pastecs’^(39)^ and ‘psych’^(40)^. Days when lunch was not eaten in the school restaurant (*n* 667) were excluded, whereas pupils who had no dietary intake reported during school lunch hours (*n* 33) were not excluded but were assigned a zero-intake at lunch. Excluding these cases did not substantially affect the mean values or statistical significance. The significance level for all statistical analyses was set at *P* < 0·05.

**Fig. 1:**
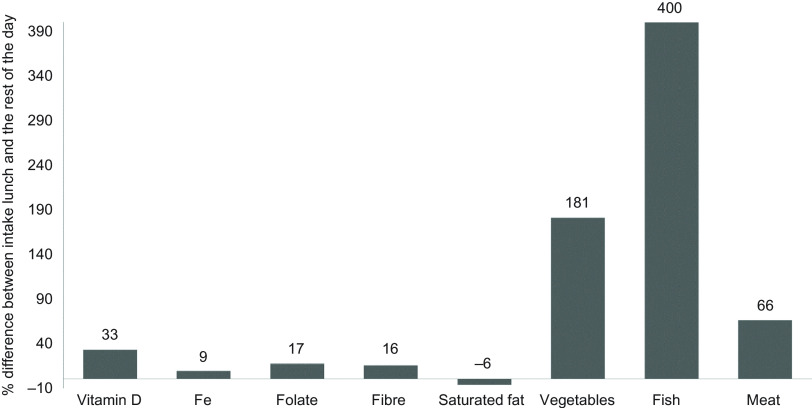
Relative (%) difference between the mean energy-adjusted intake of nutrients and foods at lunch and during the rest of the school day. A positive value means a higher intake, while a negative value means a lower intake at lunch, compared with the rest of the day.

The differences between energy-adjusted dietary intakes of nutrients and food groups and energy density, respectively, at lunch and during the rest of the day for the entire sample of pupils were analysed with mixed-effects linear regression models using the package ‘lme4’^(41)^. A dichotomous variable indicating whether the energy-adjusted dietary intake of nutrients and food groups or the energy density (respectively) were from the school lunch or from the rest of the day was treated as an independent fixed effect, while the primary sampling unit (school) was considered an independent random effect to account for the clustered nature of the data. These analyses required the data to be restructured so that each pupil’s intake was represented on two rows (one for lunch and one for the rest of the day), hence the model also included the pupils’ personal case number (ID) as an independent random effect to account for inter-individual variability. Since the nutrients and foods were measured on different scales (µg/mg or g), the relative difference (%) between the energy-adjusted intake at lunch and during the rest of the day for all nutrients and food groups was calculated to compare the magnitude of differences between intake at lunch *v*. rest of the day (Fig. 1).

*P* < 0·001 for all comparisons of energy-adjusted intake (µg, mg or g of nutrients or food group per 10 MJ) between lunch and during the rest of the day by means of mixed-effects linear regression

Differences in pupils’ dietary intake of energy, nutrients and food groups were also assessed in relation to sex and level of parental education through mixed-effects linear regression models. Here, three different dependent outcomes were assessed: (i) total daily intake of energy, nutrients and food groups, (ii) intake of energy, nutrients and food groups from lunch and (iii) percentage of daily intake of energy, nutrients and food groups consumed at lunch. The sociodemographic variables (sex, grade and parental education) were treated as independent fixed effects, while the primary sampling unit (school) was considered an independent random effect. The analysis of intake at lunch was adjusted for the pupils’ intake during the rest of the school day (independent fixed effect). This was done to remove the potential variation in the pupils’ intake at lunch explained by the pupils’ intake during the rest of the day. Controlling for weight status or area of residence did not affect parameter estimates; these variables were, therefore, excluded from the final analyses to optimise statistical power. Interactions between sex and grade were tested for each model. No significant interactions were found in the initial analyses, and the mixed linear models were, therefore, not stratified for grade.

*P*-values from the mixed-effects linear regression models were based on the analyses where the variation in independent fixed effects and that in the random effect were held constant.

## Results

### Background characteristics

A description of the study sample is presented in Table 1. The distribution of sociodemographic characteristics was similar across grade and sex. A fifth of the pupils were overweight or obese; 60 % had parents with post-secondary education; around half resided in semi-urban areas; and three-quarters were classified as Nordic. Approximately 72 % of pupils ate the school lunch on all of their (two or three) recorded school days. Boys in grade 8 had the highest proportion of pupils not reporting any dietary intake during school lunch hours on all of their recorded school days, whereas girls in grade 5 had the lowest proportion (36 *v*. 20 %). Less than 2 % did not report any dietary intake at all during school lunch hours (*n* 33).

**Table 1:** Background characteristics of all pupils (*N* 2002) according to grade and sex

Participant characteristics	All			Grade 5						Grade 8					
	*N* 2002			Girls (*n* 534)			Boys (*n* 456)			Girls (*n* 557)			Boys (*n* 455)		
	*n*	%		*n*	%		*n*	%		*n*	%		*n*	%	
Age															
Mean			13·0			11·5			11·6			14·5			14·5
sd			1·5			0·4			0·4			0·4			0·4
Overweight status*															
Overweight/obese	388	19		120	20		97	20		97	20		74	20	
Parental education															
>12 years	1208	60		339	60		271	60		343	60		255	60	
≤12 years	794	40		195	40		184	40		214	40		200	40	
Country of birth															
Nordic	1653	83		442	83		376	83		469	84		366	80	
Non-Nordic	349	17		92	17		80	17		88	16		89	20	
Area of residence															
Urban	612	31		195	40		145	30		148	30		124	30	
Semi-urban	898	44		171	30		175	40		312	60		240	50	
Rural	492	25		168	30		136	30		97	20		91	20	
School lunch reported															
On all recorded weekdays†	1438	72		428	80		352	77		369	66		289	64	

* Weight and height information was missing for twenty pupils.

† 2/2 or 3/3 d of reported dietary information; 1·6 % (*n* 33) did not eat the school lunch at all but were included in calculations.

### Intake from school lunch

Table 2 displays the mean and 95 % CI of energy intake as well as the mean and 95 % CI of absolute and energy-adjusted intakes of nutrients and food groups at lunch, together with the dietary reference values used when planning school meals^(25)^ according to grade and sex.

**Table 2:** Intake of energy, nutrients and food groups at lunch (*N* 2002)

Food/nutrient	Unit	Reference for a school lunch*	Grade 5				Grade 8			
			Girls (*n* 534)		Boys (*n* 456)		Girls (*n* 557)		Boys (*n* 455)	
			Mean	95 % CI	Mean	95 % CI	Mean	95 % CI	Mean	95 % CI
Energy	MJ	2·7–3·1	1·83	1·74, 1·92	1·97	1·87, 2·08	1·61	1·53, 1·68	2·34	2·20, 2·48
Energy density	kJ/g	NA	6·62	6·45, 6·77	6·96	6·74, 7·18	6·35	6·12, 6·58	6·98	6·70, 7·25
Vitamin D	µg	3·0	1·7	1·6, 1·8	2·0	1·8, 2·2	1·5	1·3, 1·6	2·7	2·4, 3·1
	µg/10 MJ	NA	9·2	8·7, 9·8	10·1	9·4, 10·8	8·9	8·0, 9·5	10·5	9·6, 11·3
Fe	mg	3·3–4·5	1·9	1·8, 2·0	2·0	1·9, 2·1	1·7	1·6, 1·8	2·4	2·1, 2·6
	mg/10 MJ	na	10·6	10·2, 10·9	10·3	9·9, 10·8	10·8	10·4, 11·3	10·1	9·6, 10·6
Folate	µg	60–90	66	62, 70	71	67, 76	59	55, 63	81	76, 87
	µg/10 MJ	na	378	361, 394	371	353, 389	379	361, 397	345	329, 362
Fibre	g	8–9	4·4	4·2, 4·6	4·4	4·1, 4·7	4·3	4·0, 4·5	4·9	4·5, 5·3
	g/10 MJ	NA	26·2	25·1, 27·3	23·4	22·2, 24·5	28·2	26·9, 29·5	21·9	20·7, 23·1
Saturated fat	E%	10	12·7	12·2, 13·1	13·0	12·5, 13·5	11·9	11·4, 12·4	12·0	11·5, 12·6
Vegetables	g	NA	65	60, 70	64	58, 71	65	59, 70	68	61, 75
	g/10 MJ	NA	398	367, 429	366	327, 404	477	405, 549	308	276, 341
Fish	g	NA	14	12, 16	16	13, 19	15	12, 17	28	23, 32
	g/10 MJ	NA	75	64, 86	84	70, 98	80	68, 91	99	85, 113
Red/processed meat	g	NA	30	27, 33	36	33, 40	19	17, 22	34	30, 38
	g/10 MJ	NA	156	144, 169	183	168, 198	119	108, 130	148	130, 165

* Values used for planning school meals developed by the Swedish Food Agency^(25)^: based on 30 % of daily estimated energy requirements and using a target of 30 % of daily recommended micronutrient intakes for children in grades 5 and 8, both sexes, according to the Nordic Nutrition Recommendations 2012^(26)^.

The mean energy intake at lunch was lower in all groups than the reference value. Boys in grade 8 had the highest mean energy intake at lunch, while girls in grade 8 had the lowest mean intake (2·34 *v*. 1·61 MJ). Mean absolute intakes of vitamin D, Fe and dietary fibre were lower than the reference amount in all sex and age groups, whereas the mean proportion of energy from saturated fat was higher. Girls in grade 8 also had a mean absolute folate intake below the reference amount. As for the mean absolute intake of food groups, vegetable intake at lunch was similar in all sex and age groups of pupils (64–68 g/d). All sex and age groups had a mean fish intake of around 15 g/d at lunch, except for boys in grade 8 whose mean intake was almost twice that amount. The mean consumption of red/processed meat varied by grade and sex; girls in grade 8 had the lowest mean absolute intake at lunch, while the boys in grade 5 had the highest (19 *v*. 36 g/d).

When intakes were adjusted for energy, girls in both grades had a higher mean intake at lunch than boys for all nutrients, except for vitamin D. They also had higher mean intakes of vegetables, while boys, on average, had higher intakes of fish and red/processed meat at lunch. Grade 5 pupils had higher mean intakes of saturated fat (as a percentage of energy) at lunch.

The mean energy-adjusted intake of micronutrients, dietary fibre and saturated fat, vegetables, fish and red/processed meat for the entire sample of pupils was significantly higher at lunch compared with that during the rest of the school day (online Supplemental Table 1). The relative differences (%) between mean energy-adjusted intakes of nutrients and food groups at lunch and during the rest of the day are illustrated in Fig. 1.

**Fig. 2:**
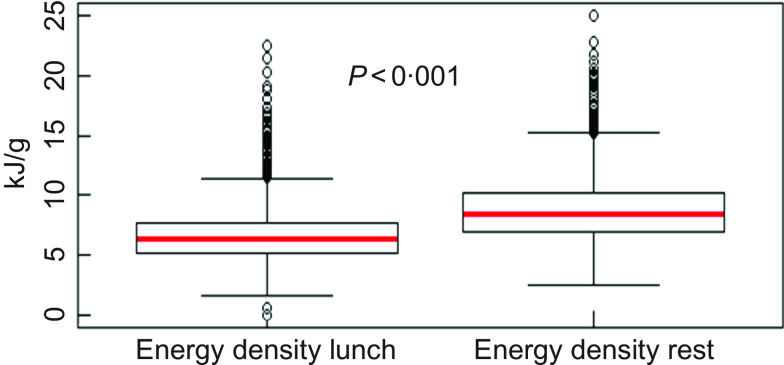
Boxplots describing the energy density (kJ/g) at lunch and during the rest of the day (*N* 2002)

The mean energy density (kJ/g) for the entire sample of pupils was significantly lower at lunch compared with the rest of the school day (online Supplemental Table 1). Quartiles and variability of energy density at lunch and during the rest of the day are illustrated in Fig. 2. A lower mean energy density at lunch *v*. during the rest of the day was also observed in all subgroups (data not shown).

### Daily intake, lunch intake and daily intake provided by the school lunch

Table 3 describes the mean energy-adjusted daily intake, mean energy-adjusted intake at lunch and the mean daily intake of energy, nutrients and food groups provided by the school lunch by sex and parental education.

**Table 3:** Total daily intake, intake at lunch and daily intake provided by lunch (%)

Nutrients/foods	Unit	Girls (*n* 1091)		Boys (*n* 911)		*P*	Parental education >12 years (*n* 1208)		Parental education ≤12 years (*n* 794)		*P*
		Mean	sd	Mean	sd		Mean	sd	Mean	sd	
Energy											
Daily intake*	MJ	7·37	2·37	8·85	3·45	<0·001	8·17	3·02	7·84	2·96	0·002
Intake at lunch†	MJ	1·72	1·02	2·16	1·34	<0·001	1·91	1·17	1·92	1·23	0·891
Daily intake provided by lunch‡	%	23	11	24	12	0·022	23	11	24	12	0·080
Vitamin D											
Daily intake*	µg/10 MJ	7·6	4·0	8·1	4·1	0·030	7·7	3·4	7·9	4·3	0·237
Intake at lunch†	µg/10 MJ	9·1	8·0	10·5	8·7	0·002	9·2	7·2	10·6	9·8	<0·001
Daily intake provided by lunch‡	%	27	18	30	19	<0·001	27	18	30	20	<0·001
Fe											
Daily intake*	mg/10 MJ	10·0	3·4	9·8	2·7	0·172	10·1	3·4	9·6	2·5	0·003
Intake at lunch†	mg/10 MJ	10·8	4·8	10·5	5·1	0·364	10·9	5·2	10·3	4·5	0·021
Daily intake provided by lunch‡	%	24	12	25	13	0·203	24	12	25	13	0·224
Folate											
Daily intake*	µg/10 MJ	335	106	324	98	0·028	338	101	316	104	<0·001
Intake at lunch†	µg/10 MJ	383	203	366	182	0·205	386	195	360	192	0·171
Daily intake provided by lunch‡	%	25	13	26	14	0·028	25	13	26	14	0·155
Fibre											
Daily intake*	g/10 MJ	23·6	7·8	21·0	6·7	<0·001	23·1	7·5	21·5	7·2	<0·001
Intake at lunch†	g/10 MJ	27·6	13·4	23·1	12·5	<0·001	26·5	13·5	24·1	13·3	0·019
Daily intake provided by lunch‡	%	25	13	25	14	0·973	25	13	25	14	0·389
Saturated fat											
Daily intake*	g/10 MJ	36·0	8·4	36·5	8·7	0·138	36·2	8·3	36·3	9·0	0·815
Intake at lunch†	g/10 MJ	33·6	14·5	34·6	14·6	0·138	33·9	14·3	34·3	14·9	0·996
Daily intake provided by lunch‡	%	22	14	24	15	0·061	22	13	24	15	0·029
Vegetables											
Daily intake*	g/10 MJ	206	146	175	135	<0·001	204	141	174	140	<0·001
Intake at lunch†	g/10 MJ	444	470	345	393	<0·001	415	402	375	747	0·465
Daily intake provided by lunch‡	%	46	29	44	30	0·110	45	28	47	32	0·044
Fish											
Daily intake*	g/10 MJ	32	47	35	54	0·406	32	47	36	54	0·028
Intake at lunch†	g/10 MJ	78	134	94	154	0·024	80	140	93	148	0·022
Daily intake provided by lunch‡	%	57	44	63	43	0·046	57	44	64	43	0·007
Red/processed meat											
Daily intake*	g/10 MJ	98	68	116	73	<0·001	102	65	112	78	0·007
Intake at lunch†	g/10 MJ	139	141	169	179	<0·001	149	147	158	148	0·875
Daily intake provided by lunch‡	%	31	29	32	28	0·169	31	28	32	30	0·586

* Mixed-effects linear regression model with daily energy or energy-adjusted nutrient or food intake as dependent variable; sex, grade and parental education as independent fixed effects; school as independent random effect.

† Mixed-effects linear regression model with energy or energy-adjusted nutrient or food intake at lunch as dependent effect; sex, grade, parental education and energy-adjusted intake during the rest of the day as independent fixed effects; school as independent random effect.

‡ Mixed-effects linear regression model with percentage of daily intake provided by lunch as dependent effect; sex, grade and parental education as independent fixed effects; school as independent random effect.

#### Sex

Compared with girls, boys had a significantly higher mean daily energy intake and higher mean daily intake of vitamin D, whereas the mean daily intake of folate and dietary fibre was significantly lower. Boys also had a significantly lower mean daily intake of vegetables but a significantly higher mean daily intake of red/processed meat.

As for lunch, boys had a significantly higher mean energy intake compared with girls (2·16 *v*. 1·72 kJ). Boys further had a significantly higher mean intake of vitamin D, and a significantly lower mean dietary fibre intake. The mean intake of vegetables at lunch was significantly lower and that of fish and red/processed meat was significantly higher in boys than in girls.

When expressing intakes from lunch as a proportion of total daily intake, the following differences reached statistical significance: the mean daily intake of energy provided by lunch was higher in boys (24 % of total daily intake) as compared with girls (23 % of total daily intake). The same was true for vitamin D, where the mean daily intake provided by lunch was higher in boys (30 % of total daily intake) as compared with girls (27 % of total daily intake). The mean daily intake of folate provided by lunch was also higher in boys (26 % of total daily intake) as compared with girls (25 % of total daily intake). Similarly, the mean daily intake of fish provided by lunch was also higher in boys (63 % of total daily intake) as compared with girls (57 % of total daily intake).

#### Parental education

Pupils of parents with lower-level education (≤12 years of education) had significantly lower mean daily intakes of energy, Fe, folate, dietary fibre and vegetables and a significantly higher mean intake of red/processed meat compared with pupils of parents with higher levels of education.

At lunch, however, there were few significant differences in mean energy intake between the two groups. Only the mean intakes of Fe and dietary fibre were significantly lower in pupils of parents with lower-level education, while the mean intakes of vitamin D and fish were higher.

When expressing intakes from lunch as a proportion of total daily intake, several differences were seen. The mean daily intake of vitamin D provided by lunch was significantly higher in pupils of parents with lower-level education (30 % of total daily intake) as compared with pupils of parents with higher levels of education (27 % of total daily intake). The same was true for saturated fat, where the mean daily intake provided by lunch was significantly higher in pupils of parents with lower-level education (24 % of total daily intake) as compared with pupils of parents with higher levels of education (22 % of total daily intake). The mean daily intake of fish provided by lunch was also significantly higher in pupils of parents with lower-level education (64 % of total daily intake) as compared with pupils of parents with higher levels of education (57 % of total daily intake). Similarly, the mean daily intake of vegetables provided by lunch was also significantly higher in pupils of parents with lower-level education (47 % of total daily intake) as compared with pupils of parents with higher levels of education (45 % of total daily intake).

## Discussion

### Main findings

In this representative sample of Swedish school children in grades 5 and 8, the school lunch accounted for – on weekdays – around a quarter of the overall energy intake; between 22 and 30 % of selected nutrient intakes; almost half of vegetable intakes; roughly two-thirds of fish intakes; and around a third of red/processed meat intakes. These findings imply that school meals make an important contribution to children’s diets on weekdays. Despite this, the goal that school meals provide approximately 30 % of daily reference values is not quite being met for certain nutrients like vitamin D, Fe and fibre. Although the nutritional quality of school meals as provided^(30)^ and consumed^(24)^ has improved, with regard to these nutrients, there is still room for further improvement.

Nonetheless, the nutrient density of the school lunch was higher, and the energy density lower, than that of the food consumed during the rest of the day, suggesting that school meals are more nutritious than meals consumed outside of school. After adjusting for differences in energy intake, boys consumed more fish and red/processed meat, but less fibre and vegetables at lunch, than girls. The daily intakes of energy, most nutrients and foods were lower for the pupils of parents with lower education compared with the pupils of parents with higher education, but when lunch intake was compared, only Fe and fibre intake was lower in this group. This suggests that school meals can play a role in compensating for lower dietary quality in the home environment.

### Interpretation/comparison to other studies

National guidelines for the planning of school meals^(25)^ are well established in Sweden, and most municipalities seem to follow these guidelines ^(42)^. In our study, nutrient density was higher, and energy density lower, at lunch compared with the rest of the weekday. Studies from the UK have also shown that school meals that follow food-based standards have a higher nutrient density than packed lunches prepared at home and consumed in school^(20,43–46)^. This stands in contrast to findings from Canada^(47)^, where the nutrient density of most studied micronutrients was lower during school hours compared with meals consumed on non-school hours in a national sample of pupils. These differences might be explained by the broad variation in adherence to school nutrition guidelines and financial prerequisites across the country^(47)^. Furthermore, the Canadian study assessed intake during school hours (09.00–14.00 hours), which could also have included intakes of energy-dense and nutrient-poor snacks. On the other hand, meals consumed during school hours provided significantly more vegetables to children as compared with meals consumed on non-school hours^(47)^. Our findings also show that the school lunch accounted for around half of pupils’ daily intake of vegetables, which is not surprising since vegetables are typically consumed at lunch and dinner rather than at breakfast. Other studies in Denmark^(48)^ and the USA^(49)^ similarly showed that meals provided in school are superior to lunches brought from or consumed at home in terms of promoting the intake of nutritious foods such as fruit, vegetables and fish.

Importantly, our results suggest that school meals in Sweden may level out some of the well-known diet-related inequalities related to education. This aligns well with findings from Norway, where the introduction of free school meals was shown to increase the intake of healthy foods, especially among socioeconomically disadvantaged pupils^(50)^. In other high-income settings like Japan and the UK, school meals have also been shown to aid in narrowing socioeconomic inequalities in dietary intake^(51–53)^. Taken together, these findings emphasise the decisive role of school lunches, as well as the importance of national nutrition standards and policies for school lunches, in providing the same prerequisites for all children to access nutritious foods, and in seeing that these meals are also consumed as intended. In contrast, differences in meat and vegetable intakes seen in the Swedish adult population^(8)^ appear to be already present in this age group. The results showed that boys had a higher intake of red/processed meat but a lower intake of vegetables and dietary fibre compared with girls both overall and at lunch specifically. Similar findings have been observed in European adolescents^(54)^ and adults^(12)^. This supports the idea of how females and males historically have created and upheld gender by consuming gender-specific foods^(55)^. Understanding the pathways that lead to differences in dietary intake between the sexes will be important when developing health promotion interventions.

The mean dietary intake of energy and nutrients at lunch did not reach 30 % of the daily energy and nutrient requirements, which is in line with previous findings on Swedish school meals^(24,30)^. Although the absolute intakes of, for example, dietary fibre, saturated fat, vitamin D and folate from the Swedish school lunch in the current study appear to be better aligned with recommendations compared with results from 2003^(24)^, there is room for further improvement. Yet, it might be challenging to draw any definite conclusions from these findings to consequences for health since current recommendations used for planning the school lunch are based on values set to cover the needs of 97–98 % of school-aged children and are, thus, not fully comparable to the mean intakes assessed here or to average dietary requirements.

In our sample, the intake of saturated fat at lunch was higher than the recommended 10 % of energy intake, which is in line with previous findings on school meals in Sweden^(24,30)^. Saturated fat intake exceeded the recommendation during the rest of the day as well, reflecting the typical Swedish diet with a high level of dairy products and red/processed meat for both adolescents^(56)^ and adults^(8)^. The target for weekly meat consumption in Sweden is 300 g of red/processed meat per week^(57)^. In our study, the school lunch contributed to around a third of the daily intake of red/processed meat. This would amount to a mean daily intake of between 72 and 101 g per school day (corresponding to 500–700 g/week) depending on the sex and age group (data not shown), by far exceeding the Swedish weekly target. This may not only have negative implications for health but also may be seen as problematic from an environmental sustainability perspective^(58)^. In a modelling study from the Netherlands, a partial replacement of animal products with plant-based foods lowered saturated fatty acid intake and increased pupils’ fibre intake^(59)^ while maintaining micronutrient intakes. Hence, there is a need to explore how the Swedish school meal system could address these challenges by serving lunches that meet both health and environmental goals.

### Strengths and limitations

Riksmaten Adolescents 2016–2017 is the second nationally representative study on dietary habits of Swedish children^(32)^ and the first to cover older children of 14–15 years. The data enabled an exploration of the significance of school lunch in relation to sex and grade and a re-assessment of school lunch intake in grade 5^(24)^ after the introduction of the new school law in 2011. For the first time, school lunch intake was also assessed in older pupils and by socioeconomic background. Another strength is that pupils’ diet was assessed using a validated dietary assessment tool that provided detailed information on all foods and drinks consumed^(32)^. A limitation is, however, that pupils were only asked to provide diet information on 2 or 3 weekdays, limiting the extrapolation to a full school week.

Under-reporting and non-reporting might have affected our results as errors in dietary data is a common problem affecting the estimates of nutrient intakes^(60,61)^. This issue might be of less relevance to the current analysis since the data used might have been subject to both over- and under-reporting^(62)^. Our analyses were further limited by having parental education as the only indicator of socioeconomic status. There are many factors such as family income and parenting styles and practices that affect children’s dietary intake^(63)^. These interactions may be complex, and there is a need to study them more carefully to avoid drawing biased conclusions regarding cause and effect^(64)^.

## Conclusions

The current study provides confirming evidence on the importance of school meals in the dietary intake of children in Sweden. The findings suggest that Swedish school meals play an important role in the overall dietary intake on weekdays, especially for the intake of vegetables and fish, which are covered by almost half and two-thirds, respectively, by the school lunch. Our results further suggest that the school meal system provides a supportive environment for the development of good dietary habits. The pupils’ dietary intake in the current study reflects the dietary patterns of the adult population, where men consume more meat and women more vegetables. Although improved since last assessed in 2003, children’s mean intakes of vitamin D, Fe and fibre are still below, and saturated fat above, the nutrient criteria for school meals. School meals have the potential to promote healthy dietary habits from a young age and to reduce sex and social inequalities in health in the long term in Sweden as well as in other contexts.

## Supplementary material

For supplementary material accompanying this paper visit https://doi.org/10.1017/S1368980020000099. click here to view supplementary material
