# Supplementary material for: The importance of school lunches to the overall dietary intake of children in Sweden: a nationally representative study
Source: Public Health Nutr. 2020 Apr 21;23(10):1705–15. doi: 10.1017/S1368980020000099 (PMC7267782; doi:10.1017/S1368980020000099)
Supplement: Supplementary file 1 [file S1368980020000099sup001.docx]

| **Nutrient/food** | **Unit** | **Intake Lunch** | | **Intake rest of the day** | |  |  |
| --- | --- | --- | --- | --- | --- | --- | --- |
|  |  | Mean | 95% CI | Mean | 95% CI | *ß1* | *P* |
| Vitamin D | µg/10MJ | 9.8 | 9.4-10.2 | 7.2 | 7.0-7.4 | -2.54 | <0.001 |
| Iron | mg/10MJ | 10.7 | 10.5-10.9 | 9.8 | 9.6-10.0 | -0.83 | <0.001 |
| Folate | µg/10MJ | 376 | 367-385 | 321 | 316-326 | -54.32 | <0.001 |
| Fiber | g/10MJ | 25.6 | 25.0-26.2 | 22.1 | 21.7-22.5 | -3.47 | <0.001 |
| SFA | g/10MJ | 34.1 | 33.5-34.7 | 36.4 | 36.0-36.8 | 2.30 | <0.001 |
| Vegetables | g/10MJ | 399 | 374-424 | 142 | 136-148 | -256.85 | <0.001 |
| Fish | g/10MJ | 85 | 79-91 | 17 | 15-19 | -68.02 | <0.001 |
| Meat | g/10MJ | 153 | 146-160 | 92 | 89-95 | -60.95 | <0.001 |
| Energy density | (kJ/g) | 6.7 | 6.6-6.8 | 8.8 | 8.6-8.9 | 2.05 | <0.001 |

**Supplementary Table 1.** Differences between the energy-adjusted dietary intake of nutrients and food groups and the energy density, respectively, at lunch and during the rest of the day for the entire sample of pupils (N=2002).

CI, confidence interval

*ß1,* Beta coefficient from mixed effects linear regression models with energy density or energy-adjusted nutrient/food group intake as dependent variable, a variable indicating the timing of the intake (at lunch vs. during the rest of the day) as independent fixed effect; school and pupil-ID as independent random effects.
